# Advisory Committee on Immunization Practices Recommended Immunization Schedule for Children and Adolescents Aged 18 Years or Younger — United States, 2018

**DOI:** 10.15585/mmwr.mm6705e2

**Published:** 2018-02-09

**Authors:** Candice L. Robinson, José R. Romero, Allison Kempe, Cynthia Pellegrini, Peter Szilagyi

**Affiliations:** ^1^Immunization Services Division, National Center for Immunization and Respiratory Diseases, CDC; ^2^University of Arkansas for Medical Sciences, Little Rock; ^3^Arkansas Children's Hospital, Little Rock; ^4^Department of Pediatrics, University of Colorado Anschutz Medical Campus, Denver; ^5^March of Dimes, Washington, D.C.; ^6^Department of Pediatrics, University of California Los Angeles.

In October 2017, the Advisory Committee on Immunization Practices (ACIP) approved the Recommended Immunization Schedule for Children and Adolescents Aged 18 Years or Younger — United States, 2018. The 2018 child and adolescent immunization schedule summarizes ACIP recommendations, including several changes from the 2017 immunization schedules, in three figures and footnotes to the figures. These documents can be found on the CDC immunization schedule website (https://www.cdc.gov/vaccines/schedules/index.html). These immunization schedules are approved by ACIP (https://www.cdc.gov/vaccines/acip/index.html), the American Academy of Pediatrics (https://www.aap.org), the American Academy of Family Physicians (https://www.aafp.org), and the American College of Obstetricians and Gynecologists (https://www.acog.org). Health care providers are advised to use the figures and the footnotes together. The full ACIP recommendations for each vaccine, including contraindications and precautions, can be found at https://www.cdc.gov/vaccines/hcp/acip-recs/index.html. Providers should be aware that changes in recommendations for specific vaccines can occur between annual updates to the childhood/adolescent immunization schedules. If errors or omissions are discovered within the child and adolescent schedule, CDC posts revised versions on the CDC immunization schedule website.*

Printable versions of the 2018 immunization schedules for children and adolescents aged 18 years or younger and ordering instructions for laminated versions and easy-to-read versions for parents also are available at the immunization schedule website.

For further guidance on the use of each vaccine included in the schedules, including contraindications and precautions, health care providers are referred to the respective ACIP vaccine recommendations.

## Changes in the 2018 Child and Adolescent Immunization Schedule

Changes in the 2018 immunization schedules for children and adolescents aged 18 years or younger include new or revised ACIP recommendations for poliovirus (*1*), influenza (*2*), and measles, mumps, and rubella vaccines (*3*), and clarification of the recommendations for rotavirus and pneumococcal vaccines.

## Changes Affecting Multiple Portions of the Schedule

Mention of MenHiberix (Hib-MenCY) vaccine has been removed from Figure 1 and Figure 2 and the relevant footnotes (Hib and meningococcal A,C,W,Y). Manufacturing of MenHibrix has been discontinued in the United States and all available doses have expired.

**Cover Page.** Changes to the 2018 figure from the 2017 schedule^†^ are as follows:

- A table was added outlining vaccine type, abbreviation, and brand names for vaccines discussed in the child/adolescent immunization schedule.

**Figure 2**. Changes to the 2018 figure from the 2017 schedule are as follows:

- The maximum ages for the first and last doses in the rotavirus vaccination series were added to the rotavirus vaccine row.
- The inactivated poliovirus vaccine rows were edited to clarify the catch-up recommendations for children 4 years of age and older.

**Figure 3.** Changes to the 2018 figure from that in the 2017 schedule are as follows:

- A reference was added to the HIV column of the figure. The reference provides additional information regarding HIV laboratory parameters and use of live vaccines.
- Within the pneumococcal conjugate row, stippling was added to heart disease/chronic lung disease, chronic liver disease, and diabetes columns to clarify that, in some situations, an additional dose of vaccine might be recommended for children with these conditions.

**Footnotes.** The footnotes are presented in a new simplified format. The goal was to remove unnecessary text, preserve all pertinent information, and maintain clarity. This was accomplished by a transition from complete sentences to bullets, removal of unnecessary or redundant language, and formatting changes. In addition to this overall simplification, content changes were made as follows:

- The Hepatitis B vaccine (HepB) footnote was revised to include information regarding vaccination of <2,000-g infants born to hepatitis B virus surface antigen (HBsAg)–negative mothers.
- The poliovirus vaccine footnote was revised to include updated guidance for persons who received oral poliovirus vaccine as part of their vaccination series.
- The influenza vaccine footnote has been updated to indicate that live attenuated influenza vaccine (LAIV) should not be used during the 2017–2018 influenza season. A reference link to the 2017–2018 season influenza recommendations has been added.
- The measles, mumps, and rubella vaccine (MMR) footnote was updated to include guidance regarding the use of a third dose of mumps virus–containing vaccine during a mumps outbreak.
- The meningococcal vaccine footnote has been edited to create separate footnotes for MenACWY and MenB vaccines.

## Footnotes

* CDC encourages organizations that previously have relied on copying the schedules to their websites instead to use syndication as a more reliable method for displaying the most current and accurate immunization schedules on an organization's website. Use of content syndication requires a one-time step that ensures an organization’s website displays current schedules as soon as they are published or revised; instructions for the syndication code are available on CDC’s website (https://www.cdc.gov/vaccines/schedules/syndicate.html). CDC also offers technical assistance for implementing this form of content syndication (e-mail request to ncirdwebteam@cdc.gov).

† Past immunization schedules are available at https://www.cdc.gov/vaccines/schedules/past.html.
